# Anaconda: AN automated pipeline for somatic COpy Number variation Detection and Annotation from tumor exome sequencing data

**DOI:** 10.1186/s12859-017-1833-3

**Published:** 2017-10-03

**Authors:** Jianing Gao, Changlin Wan, Huan Zhang, Ao Li, Qiguang Zang, Rongjun Ban, Asim Ali, Zhenghua Yu, Qinghua Shi, Xiaohua Jiang, Yuanwei Zhang

**Affiliations:** 1 Molecular and Cell Genetics Laboratory, The CAS Key Laboratory of Innate Immunity and Chronic Diseases, Hefei National Laboratory for Physical Sciences at Microscale, School of Life Sciences, CAS Center for Excellence in Molecular Cell Science, University of Science and Technology of China, Hefei, Anhui 230027 China; 2 Reproductive Medicine Center of Jinghua Hospital, USTC-Shenyang Jinghua Hospital Joint Center of Human Reproduction and Genetics, Shenyang, Liaoning 110005 China; 3 School of Information Science and Technology, University of Science and Technology of China, Hefei, 230027 China

**Keywords:** Copy number variation, Exome sequencing, Functional analysis, Cancer

## Abstract

**Background:**

Copy number variations (CNVs) are the main genetic structural variations in cancer genome. Detecting CNVs in genetic exome region is efficient and cost-effective in identifying cancer associated genes. Many tools had been developed accordingly and yet these tools lack of reliability because of high false negative rate, which is intrinsically caused by genome exonic bias.

**Results:**

To provide an alternative option, here, we report Anaconda, a comprehensive pipeline that allows flexible integration of multiple CNV-calling methods and systematic annotation of CNVs in analyzing WES data. Just by one command, Anaconda can generate CNV detection result by up to four CNV detecting tools. Associated with comprehensive annotation analysis of genes involved in shared CNV regions, Anaconda is able to deliver a more reliable and useful report in assistance with CNV-associate cancer researches.

**Conclusion:**

Anaconda package and manual can be freely accessed at http://mcg.ustc.edu.cn/bsc/ANACONDA/.

**Electronic supplementary material:**

The online version of this article (10.1186/s12859-017-1833-3) contains supplementary material, which is available to authorized users.

## Background

Copy number variations (CNVs) are the main genetic structural variations in human cancer genome [1–4]. Accurate inference of CNVs is necessary for identifying cancer-causing genes, and has been of long-standing interest in cancer-focused studies for investigating rules of tumor progression [5–7]. Meanwhile, the advent of next-generation sequencing (NGS) has dramatically furthered our understanding of human diseases with an unprecedented depth, as it allows high-throughput profiling of human genome in nucleotide resolution. Compared to whole-genome sequencing (WGS), whole-exome sequencing (WES) only captures and sequences exonic regions (referred as targets) and allows relatively higher coverage given at the same cost. As always, high efficiency comes with limitations. CNV detection in WES data is likely to has a high false negative rate as a consequence of the uneven distribution of exons across the cancer genome [8].

According to the recent reviews [8, 9], the existed tools showed their specialties in detecting CNVs. However, when analyzing clinical sequencing data, the performances of current CNV detecting algorithms are far from satisfactory. In clinical settings, integrative power in CNV detection is likely to achieve the most stable performance [10]. It should contain following features: 1) Adopted different strategies, current tools show significant divergence in performance. For instance, ADTEx is most likely to detect medium-size CNVs [11], while EXCAVATOR tends to identify CNVs between 1 Mb and 100 Mb [9]. Thus, a new tool that incorporates different methods can deliver a more comprehensive detection. 2) Parameters of the integrative approach should be extensive and easy to modify. CNV detection results are greatly related to parameter settings [8], thus optimal performance of each included method requires the easy modification of parameters. 3) As high precision for CNV detection could not be easily achieved by simply adopting the multiple algorithms, broad annotations should be conducted as a guidance for users in the analysis of datasets.

To these ends, we developed Anaconda (*AN A*utomated pipeline for somatic *CO*py *N*umber variation *D*etection and *A*nnotation from tumor exome sequencing data), which successfully satisfied the requirements: 1) Anaconda is designed to be compatible with ease of use and rich features. Running Anaconda only needs one single command “*./bin/ANACONDA /path/to/configfile*”. Users could easily modify the parameters in config file. Detailed explanation of each parameter could be found at Anaconda website. 2) While utilizing different strategies, users need to locally install and configure the respective running environment for different tools, which sometime is highly challenging for general users. After downloading Anaconda package, by single command “*./install*”, Anaconda would automatically install and configure the running environment. When running, Anaconda will extract the detected CNV results of the user-selected methods. Consensus results are also generated if CNVs called by multiple methods. 3) To further explore the biological functions beneath shared CNVs, Anaconda can also conduct annotation analysis for the genes that are involved in all CNV regions called by selected tools. Thus, we believe that Anaconda could assist users in a comprehensive and effective manner with their CNV-related projects.

## Implementation

### Choice of methods

At present, lots of calling tools are available and these tools exhibited their specialties in CNV calling [8–10, 12]. To integrate the different tools into a single package, several factors weighs heavily in our consideration: 1) Efficiency: the efficiency of Anaconda depends on the performance of the included methods. Based on previous report [9], EXCAVATOR, ADTEx and Control-FREEC ranked in the top 3 for processing duration. Tested on our in-house input, ExomeCNV performed slightly slower than EXCAVATOR and ADTEx but out-performed than Control-FREEC. 2) Precision: we identified the precision of each tool based on existing comparisons, especially focus on the comparison conducted on clinical data. When setting SNP array results as control, previous report compared the performance of 6 tools on two major datasets: ADTEx and EXCAVATOR showed better performances owing to their high precision and sensitivity [9]. 3) Input: unified input format will facilitate the combination of different methods. Most caller tools, such as ADTEx, EXCAVATOR, ExomeCNV and Control-FREEC, allow BAM input. Though ERDS-pe also allows BAM input, the required single-nucleotide variation information (VCF format), limited its practicability. Additionally, the tools revealed their preference on CNV size: EXCAVATOR often recognizes larger CNVs, ADTEx tends to detect medium-size CNVs, while ExomeCNV and Control-FREEC are in favor of smaller CNVs [9]. Therefore, Anaconda integrates 4 algorithms: Control-FREEC [13], ADTEx [11], EXCAVATOR [14] and ExomeCNV [15], other tools will be incorporated to Anaconda in future.

Fundamental framework of Anaconda is constructed with Shell. Unix-like systems, R3.0+, Jdk8+, gcc and g++ are required before installing Anaconda. After fulfilling all prerequisites, users could simply run a single command “*./install*” at the Anaconda unzipped folder to install Anaconda.

### Workflow

For convenience of users during setting the parameters, Anaconda prepared a specific config file, at which users could determine the following options: 1) softwares used for CNV detection, 2) paths for input files and output results; 3) gene coverage in CNV regions; 4) minimal called methods in considering CNV as a common CNV; 5) parallel threads as well as all specific parameters for each selected tool. After the setting progress, users could simply run a command “*./bin/ANACONDA /path/to/configfile*” to process their data. We highly recommend users to access Internet when use Anaconda for the first time, because Anaconda would double-check and download the necessary packages automatically.

**Fig. 1:**
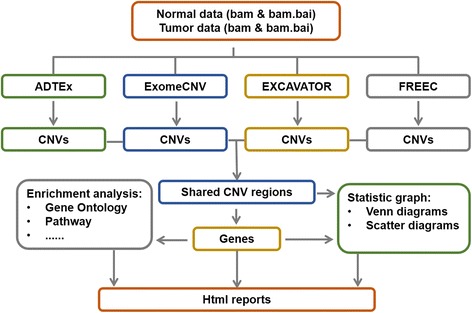
Overall workflow of Anaconda

Anaconda takes paired tumor and normal bam files, genome reference fasta file, exome bed file as input, and output detected CNVs and their annotations. Human genome (hg18 and hg19) fasta file and exome bed file can be downloaded from Anaconda website. Workflow of Anaconda is shown in Fig. 1. The pipeline contains five steps: 1) configure the running environment; 2) detect somatic CNVs by assigned tools; 3) extract the intersection of detected CNVs; 4) retrieve and annotate genes located within called CNVs; 5) generate a HTML-based report including all the analyzed results.

### General analysis for callers

For CNVs called by specific tool, Anaconda draws plot of gain and loss CNVs on every chromosome using R (Additional file 1: Figure S1A), and calculates overall loss and gain of the CNV quantity. Detailed results of CNVs are presented in tables including chromosome, exon start, exon end and copy number information (Additional file 1: Figure S1B).

Venn diagrams are drawn to show the intersection of called CNVs by selected tools and genes involved in CNV regions (Additional file 1: Figure S1C). Detailed CNV intersection results are showed in tables, including CNV position, copy number quantity, caller information and shared number information (Additional file 1: Figure S1D). Anaconda also provides additional coverage and detailed information for the genes involved in called CNV regions.

### Shared CNVs and genes

Method that Anaconda determines shared CNV region and genes can be seen at Additional file 2: Figure S2. At first, Anaconda gathers all merged CNV reads called by selected tools, maps them with reference genome and divides them as unique-caller reads, double-caller reads, triple-caller reads and tetrad-caller reads. Mapping gene to called CNV region is based on gene coverage. Our default coverage value is 0.7, i.e. if 70% of gene sequence is located inside this CNV, this gene will be retrieved with caller information. Gene coverage value could be modified at Anaconda config file.

### Functional annotation

To reveal gene function in called CNV regions, Anaconda annotates these genes with Gene Ontology (GO), Online Mendelian Inheritance in Man (OMIM), Clusters of Orthologous Groups (COG), Pathway, Protein domain and terms (Additional file 3: Figure S3). All term information are downloaded from Database for Annotation, Visualization and Integrated Discovery (DAVID) V6.8 [16] and Kyoto Encyclopedia of Genes and Genomes (KEGG) [17]. Anaconda applies fisher’s exact test to generate *P*-value for all variants enriched to respective terms. After assigning annotation categories, detailed table is provided to present annotation results. On each annotation page, search module and data sort function is equipped for users with specific commands. For instance, users could click the sort icon by *P*-value column to sort the *P*-value of all the terms in a low to high or high to low manner.

## Results and discussion

To evaluate the performance gain of Anaconda, we used thirteen simulated samples to evaluate the performance of Anaconda and the individual tool. Each simulated sample contains ten CNVs regions range from one to twenty copies (the size ranges from 500 kb to 4.5 Mb). The definition of true positives (TP), true negatives (TN), false positives (FP) and false negatives (FN) were described in our previous work [18]. The statistical measures of true positive rate (TPR), false discovery rate (FDR) and precision were used to evaluate the performance of individual or combined algorithms. Compared with results from individual software, the approach of integration of different algorithms has more stable performance. The false discovery rate was reduced from 0.0417%–17.7877% to 0.0011%–0.4854%, and the precision was increased from 82.21%–99.96% to 99.51%–100.00% (Additional file 4: Table S1).

**Fig. 2:**
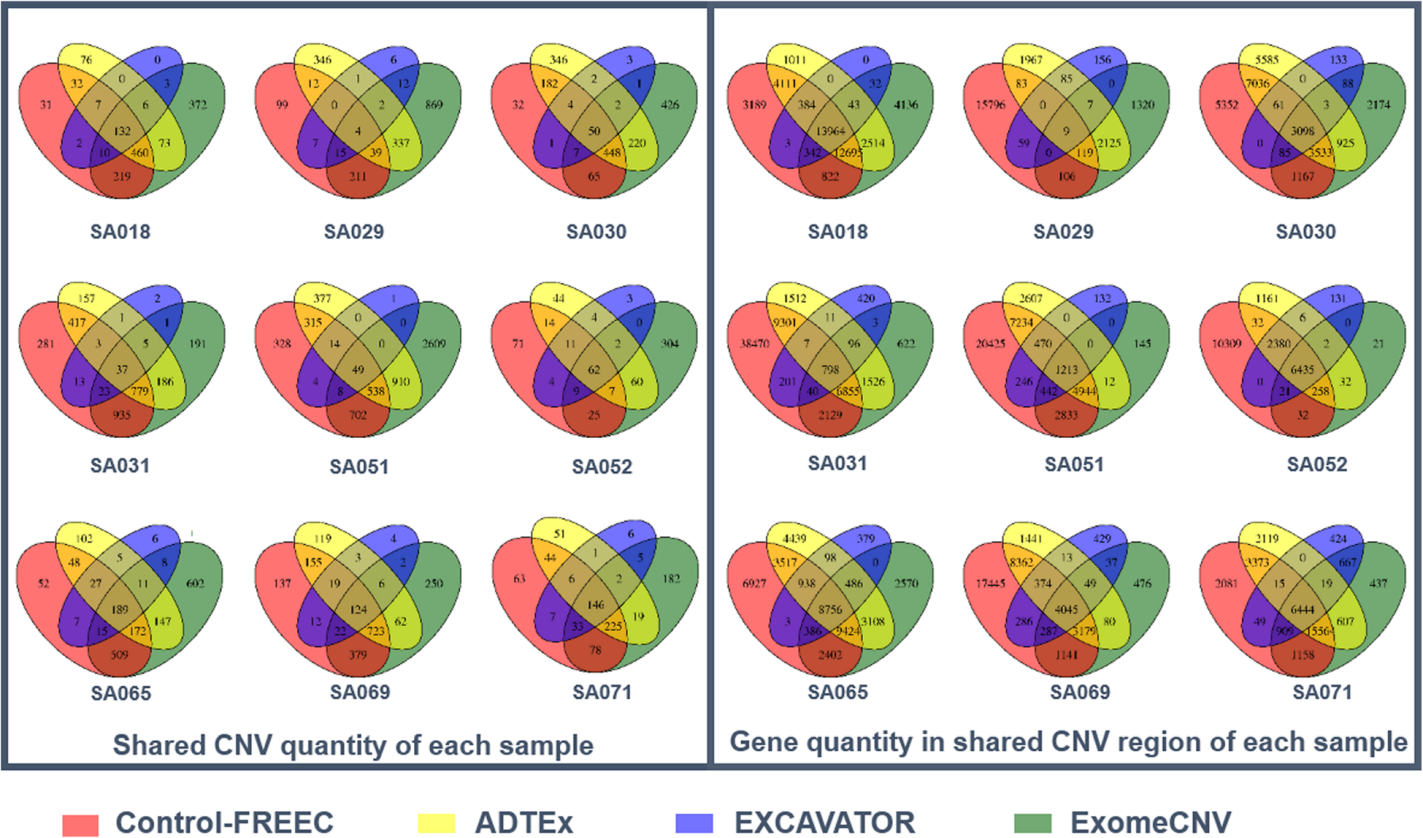
Shared CNVs and genes called by four tools

To demonstrate the high practicability of Anaconda in detecting and annotating somatic CNVs, and to evaluate the function it presents, we applied Anaconda to analyze a tumor WES dataset downloaded from European Genome Phenome Archive (EGA) with accession number EGAS00001000132. We randomly picked 9 samples, SA018, SA029, SA030, SA031, SA051, SA052, SA065, SA069 and SA071 from this dataset. During the analysis, all samples are conducted with the default parameters. For each sample, all the four calling methods, Control-FREEC, ADTEx, EXCAVATOR and ExomeCNV were applied to call CNVs from WES data. Venn diagrams were plotted (Fig. 2) to compare the overlapping results of called CNVs and genes in called CNV regions.

**Fig. 3:**
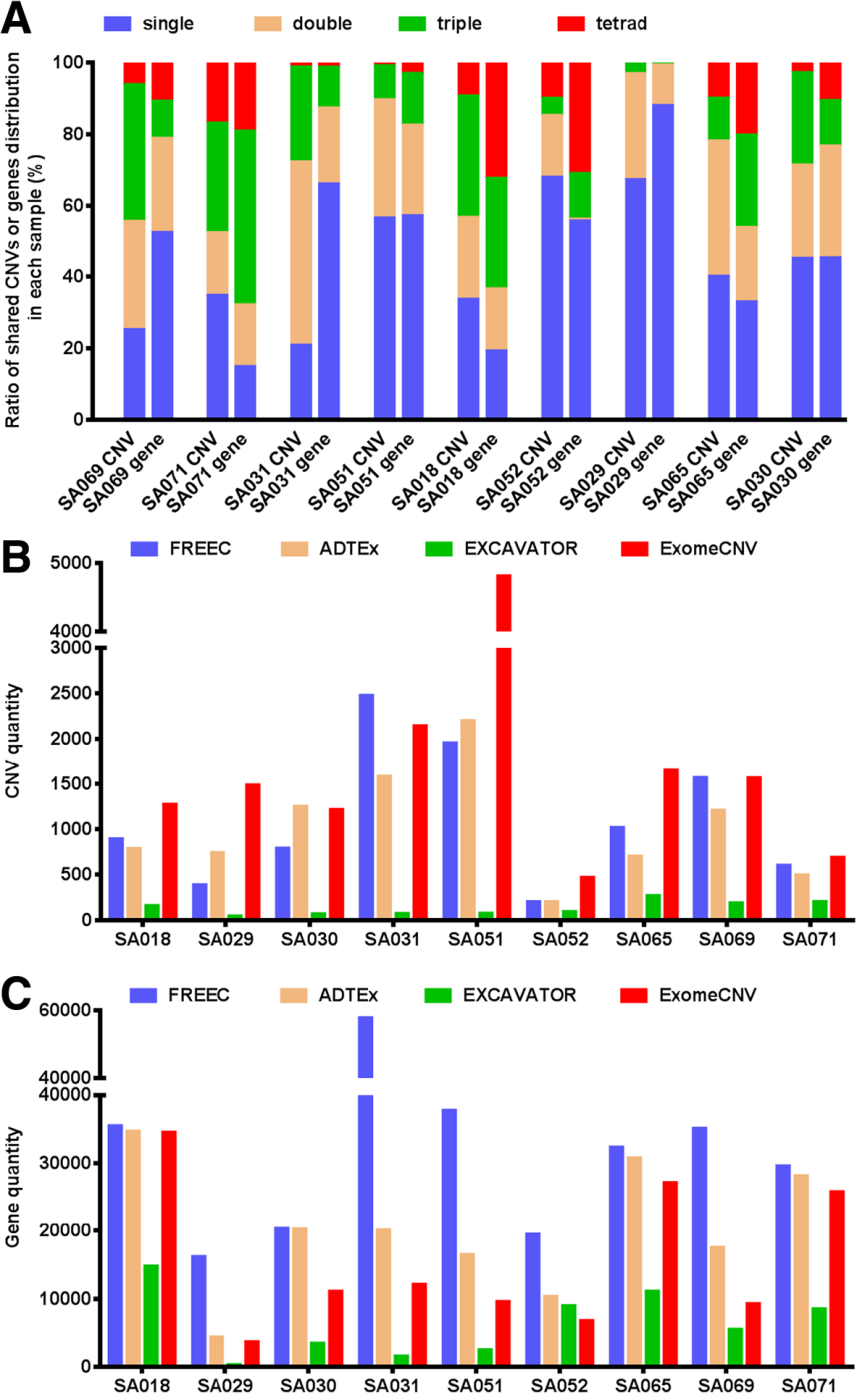
Distribution of shared reads and genes as well as the performance evaluation of each tool. **a**. Distribution of differently shared reads and the genes in according CNV regions. **b**. CNV quantity called by different tools in each sample. **c**. Gene quantity in the CNV region called by different tools in each sample

Distribution of called CNVs and genes are shown in Fig. 3a. Shared CNV regions by 4 callers (tetrad-caller reads) are significantly decreased, ranging from 0.2% in SA029 to 16.8% in SA071. Gene distribution in tetrad-caller read regions is relatively higher than triple, double or single caller reads, as the percentages of gene quantity in tetrad-caller region, over the quantity of all genes is two times higher than the percentage of tetrad-caller CNVs quantity over all CNVs. CNVs called by each tool (Fig. 3b) and gene quantity in accordance with the CNV regions (Fig. 3c) demonstrated great divergence of the performance of each tool. For example, ExomeCNV is likely to call more CNVs than others. CNV regions called by Control-FREEC tend to cover more genes. ADTEx shows a moderate performance in calling CNVs as well as the distribution of genes in its called CNV regions. EXCAVATOR called the least in quantity of CNV regions. These regions share the relatively higher overlapping rate with other tools. For example, in SA018, 82.5% of CNVs called by EXCAVATOR are also the callers by other three tools.

## Conclusion

Anaconda is an integrative tool in the detection and annotation of CNVs from whole-exome sequencing data. Utilizing four published tools, Anaconda is able to detect CNVs in a comprehensive manner. Ease in installation and application, Anaconda could satisfy the biologist’s demands in data process. Additionally, pervasive annotation of genes in called CNV regions could serve as a second opinion during the analysis of datasets, compensating the low preciseness caused by the unevenly distributed sequence data. In all, we believe Anaconda could be of great help for users with their CNV-related cancer research.

## Availability and requirements

The package and manual for Anacond a can be freely accessed at http://mcg.ustc.edu.cn/bsc/ANACONDA/. Tools integrated in Anaconda could be find in the referenced articles. WES test dataset is downloaded from European Genome Phenome Archive (EGA) with accession number EGAS00001000132.

## Additional files

Additional file 1: Figure S1. General analysis of Anaconda. (TIFF 1228 kb)

Additional file 2: Figure S2. Anaconda detected shared CNV regions and genes. The region is considered as unique-caller read, only called by ADTEx; b region is considered as double-caller read, called by ADTEx and EXCAVATOR; c region is considered as triple-caller read, called by EXCAVATOR, Control-FREEC and ADTEx; d region is considered as tetrad-caller read, called by all four tools. Mapping gene to CNV region is based on gene sequence coverage in CNV region. (TIFF 70 kb)

Additional file 3: Figure S3. Functional annotations of Anaconda. (TIFF 395 kb)

Additional file 4: Table S1. Evaluation of performance gain of Anaconda. (DOCX 16 kb)

## Abbreviations

Anaconda: AN Automated pipeline for somatic COpy Number variation Detection and Annotation from tumor exome sequencing data
CNV: Copy number variation
NGS: Next generation sequencing
WES: Whole exome sequencing
WGS: Whole genome sequencing
