# Supplementary figures and images for: Anaconda: AN automated pipeline for somatic COpy Number variation Detection and Annotation from tumor exome sequencing data
Source: BMC Bioinformatics. 2017 Oct 3;18:436. doi: 10.1186/s12859-017-1833-3 (PMC5627484; doi:10.1186/s12859-017-1833-3)

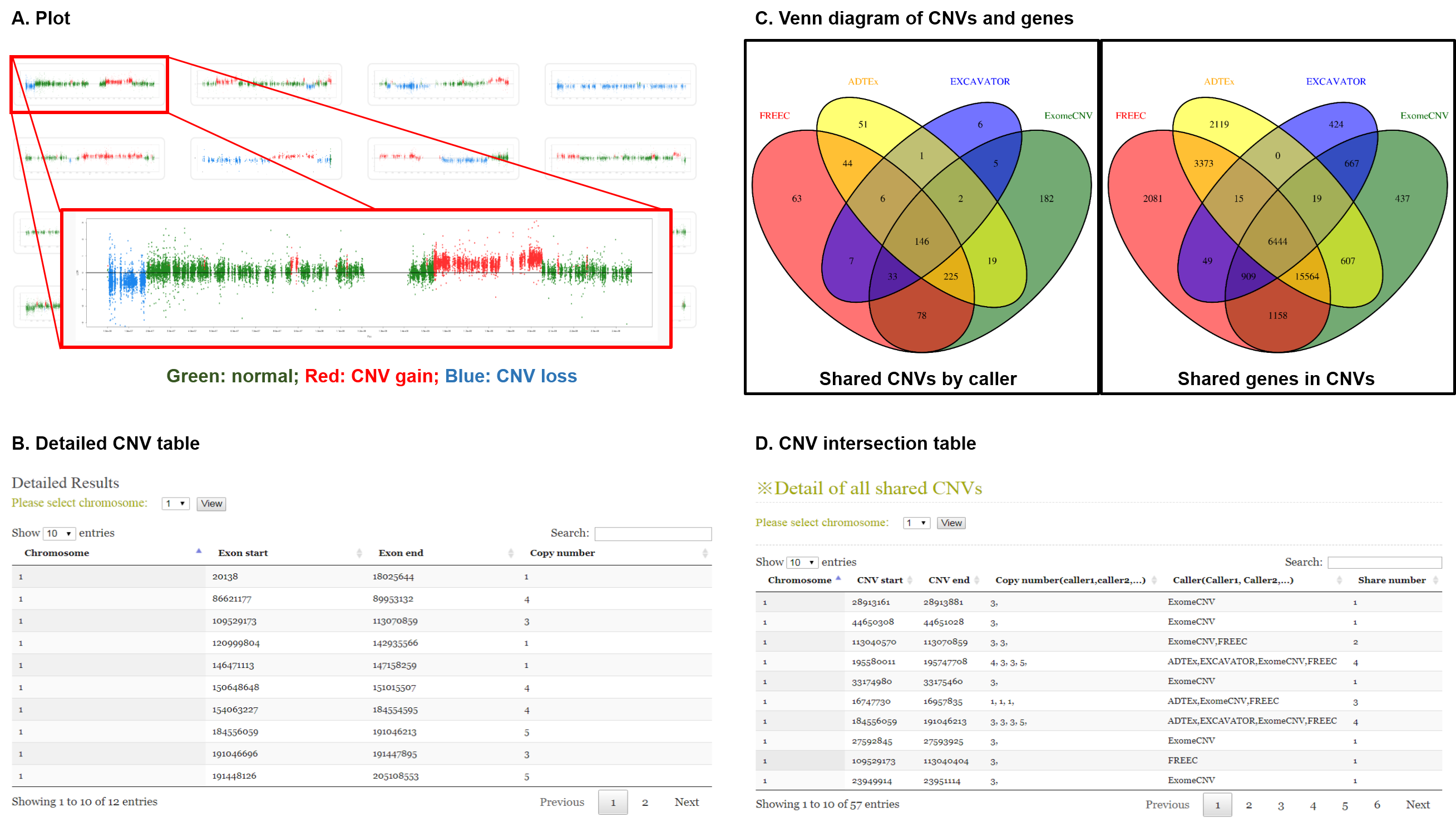

Supplement: Supplementary file 1 — General analysis of Anaconda. (TIFF 1228 kb) [file 12859_2017_1833_MOESM1_ESM.tif]

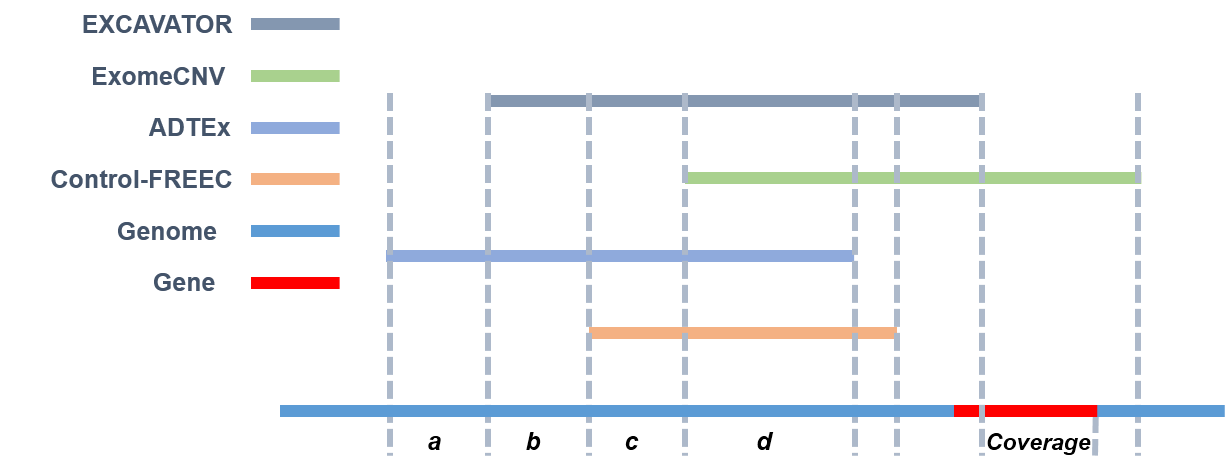

Supplement: Supplementary file 2 — Anaconda detected shared CNV regions and genes. The region is considered as unique-caller read, only called by ADTEx; b region is considered as double-caller read, called by ADTEx and EXCAVATOR; c region is considered as triple-caller read, called by EXCAVATOR, Control-FREEC and ADTEx; d region is considered as tetrad-caller read, called by all four tools. Mapping gene to CNV region is based on gene sequence coverage in CNV region. (TIFF 70 kb) [file 12859_2017_1833_MOESM2_ESM.tif]

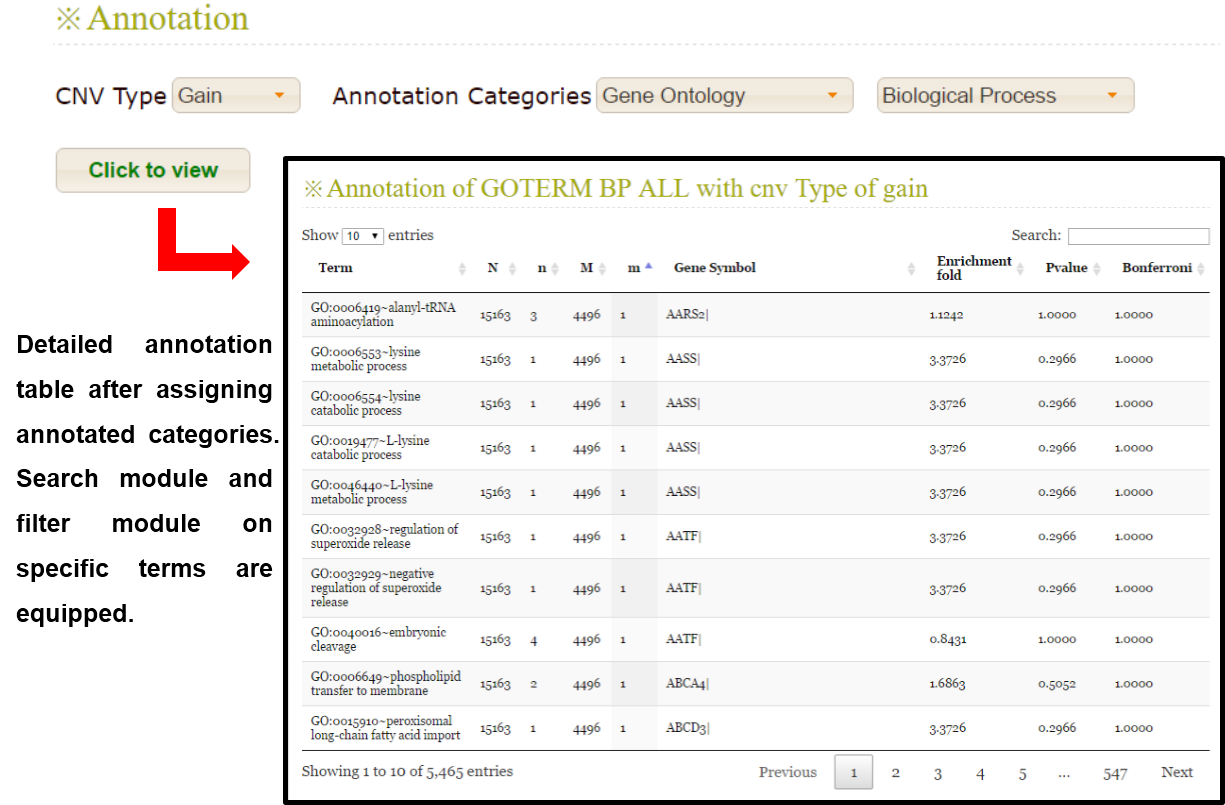

Supplement: Supplementary file 3 — Functional annotations of Anaconda. (TIFF 395 kb) [file 12859_2017_1833_MOESM3_ESM.tif]
