# Supplementary material for: Anaconda: AN automated pipeline for somatic COpy Number variation Detection and Annotation from tumor exome sequencing data
Source: BMC Bioinformatics. 2017 Oct 3;18:436. doi: 10.1186/s12859-017-1833-3 (PMC5627484; doi:10.1186/s12859-017-1833-3)
Supplement: Supplementary file 4 — Evaluation of performance gain of Anaconda. (DOCX 16 kb) [file 12859_2017_1833_MOESM4_ESM.docx]

Additional file 4: Table S1 Evaluation of performance gain of Anaconda

| **Software** | **Software number** | **TP** | **FP** | **FN** | **TPR** | **FDR** | **Precision** |
| --- | --- | --- | --- | --- | --- | --- | --- |
| ADTEx | 1 | 23917 | 241 | 3910 | 0.8595 | 0.9982% | 99.00% |
| FREEC | 1 | 26756 | 5789 | 1071 | 0.9615 | 17.7877% | 82.21% |
| EXCAVATOR | 1 | 13130 | 106 | 14697 | 0.4718 | 0.8072% | 99.20% |
| ExomeCNV | 1 | 21237 | 8 | 6590 | 0.7632 | 0.0417% | 99.96% |
| ADTEx+ExomeCNV | 2 | 20458 | 4 | 7369 | 0.7352 | 0.0195% | 99.98% |
| ADTEx+EXCAVATOR | 2 | 13091 | 2 | 14736 | 0.4704 | 0.0191% | 99.98% |
| ADTEx+FREEC | 2 | 23371 | 114 | 4457 | 0.8398 | 0.4854% | 99.51% |
| ExomeCNV+EXCAVATOR | 2 | 13103 | 1 | 14724 | 0.4709 | 0.0098% | 99.99% |
| ExomeCNV+FREEC | 2 | 20291 | 3 | 7536 | 0.7292 | 0.0179% | 99.99% |
| EXCAVATOR+FREEC | 2 | 13128 | 1 | 14700 | 0.4718 | 0.0125% | 99.99% |
| ADTEx+FREEC+EXCAVATOR | 3 | 13090 | 0 | 14737 | 0.4704 | 0.0011% | 100.00% |
| ADTEx+FREEC+ExomeCNV | 3 | 20010 | 0 | 7818 | 0.7191 | 0.0021% | 100.00% |
| ADTEx+ExomeCNV+EXCAVATOR | 3 | 13065 | 0 | 14762 | 0.4695 | 0.0016% | 100.00% |
| FREEC+EXCAVATOR+ExomeCNV | 3 | 13102 | 0 | 14726 | 0.4708 | 0.0033% | 100.00% |
| ADTEx+FREEC+EXCAVATOR+ExomeCNV | 4 | 13064 | 0 | 14764 | 0.4695 | 0.0011% | 100.00% |
